# The impact of body mass index on prostate cancer: An updated systematic review and meta-analysis

**DOI:** 10.1097/MD.0000000000030191

**Published:** 2022-11-11

**Authors:** Nikolaos Tzenios, Mary E. Tazanios, Mohamed Chahine

**Affiliations:** a Public Health and Medical Research, Charisma University, Grace Bay, Turks and Caicos Islands, Train to Teach in Medicine, Department of Postgraduate Medical Education, Harvard Medical School, Boston, Massachusetts. Doctor of Health Sciences Candidate, MCPHS University, Boston, MA, USA; b Clinical Research, TRG GEN+, Beirut, Lebanon; c Biological and Chemical Technology, International Medical Institute, Kursk State Medical University, Kursk, Russian Federation.

**Keywords:** body mass index, obesity, overweight, prostate cancer, risk factor

## Abstract

Increasing evidence suggested obesity was associated with the risk of prostate cancer. Also, the association between prostate cancer risk and obesity has received much attention in recent years, but the results are still unclear. Therefore, the current systematic review and meta-analysis aimed to evaluate the impact of body mass index (BMI) on prostate cancer.

**Methods:**

We systematically searched PubMed, Google Scholar, Scopus and Cochrane databases with the appropriate key terms to identify the eligible articles related to the impact of BMI on prostate cancer. The Newcastle-Ottawa checklist was used for the quality assessment of studies, and the meta-analysis was carried out using Review Manager 5.3.

**Results:**

The present review includes 23 studies that fulfilled the criteria for inclusion. In the meta-analysis, a significant difference was observed between the obese and normal weight (*P* < .001) and 54% of obese has a risk compared to normal weight. Heterogeneity between the fifteen studies was high (*I*^2^ = 100%). Test for overall effect: Z = 8.77 (*P* < .001) (odds ratio [OR] = 0.32 confidence interval [CI]: 0.25–0.42). However, there was no significant difference observed between the overweight and normal weight (*P* = .75). Heterogeneity between the fifteen studies is high (*I*^2^ = 100%).

**Conclusion:**

Prostate cancer is a common malignancy that poses a threat to the health of men. Obesity is associated with a higher risk of death from prostate cancer based on the findings of the included studies. Furthermore, wherever possible, the impact of weight change on prostate cancer patient mortality should be investigated.

## 1. Introduction

In the United States, obesity is a major public health threat, affecting more than 30% of individuals.^[1]^ Obesity rates have more than doubled globally since 1980. Psychological, neuroendocrine, genetic and environmental factors all have a role in the development of obesity.^[2]^ Obesity is still uncommon in Asian countries such as Japan and Korea, with less than 10% of the population obese, although it has become more common in the recent decade.^[2,3]^ However, recent major investigations have revealed that this link is unclear.^[4]^ Other recognized factors linked to prostate cancer mortality, such as advanced age, a family history of cancer, and ethnicity,^[5]^ are unchangeable. Obesity should be included as a prognosis factor since it is a potentially modifiable factor. Body mass index (BMI) has been associated with many cancers. According to GLOBOCAN 2018, prostate cancer is the third most prevalent cancer in both men and women, as well as the second most common cancer in males worldwide.^[6,7]^ Obesity at a young age delays puberty and may lead to a lower lifetime exposure to insulin-like growth factor 1, which may influence prostate cancer development later in life.^[8,9]^ Elevated lipid levels and lipid signaling, inflammatory responses, insulin resistance, and adipokines have all been proposed as pathways to explain the association between cancer and obesity. However, it remains unclear how the convergence of these pathways drives obesity-linked cancer.^[10]^ Although the advent of prostate specific antigen (PSA) testing has resulted in earlier prostate cancer detection, its significance in lowering prostate cancer-specific mortality is significantly less certain.^[11]^ The impact of potential modifiers of PSA levels, with particular attention focused on obesity, may be represented in the contradictory findings of screening trials. Obese men have consistently lower PSA levels in their serum samples than non-obese men.^[12,13]^

Obesity may thus have an opposite effect on the incidence of prostate cancer risk in the early stages, depending on the type of prostate cancer. Low testosterone levels in obese individuals could be one of the underlying causes of this inverse relationship between obesity and localized prostate cancer. Due to a reduction in luteinizing hormone pulse amplitude and serum luteinizing hormone levels, obese men have a decreased concentration of free testosterone.^[14]^ A meta-analysis of 13 studies of advanced prostate cancer and 12 studies of localized prostate cancer found that localized prostate cancer had an inverse linear link with advanced prostate cancer and BMI had a positive linear link with BMI.^[15]^ Recently, a meta-analysis by Izquierdo et al^[16]^ reported that obesity was significantly associated with increased specific mortality of prostate cancer. Despite multiple studies suggesting a link between obesity and prostate cancer outcomes, r, several investigations failed to discover profoundly poor prostate cancer outcomes in a group with a high BMI.^[17,18]^ Therefore, the current updated systematic review and meta-analysis was performed to identify the impact of BMI on prostate cancer.

## 2. Methodology

### 2.1. Study design

The Preferred Reporting Items for Systematic Reviews and Meta-Analyses guidelines were used for conducting this systematic review and meta-analysis.^[16]^

### 2.2. Search strategy

A literature search was conducted using the relevant keywords and phrases in the following databases: Google Scholar, PubMed, Scopus, and Cochrane. All the published articles up to November 30^th^, 2021 were included in this review. Different types of keywords were be used for the search strategies such as “body mass index,” OR “BMI,” OR “obesity,” OR “overweight,” OR “weight,” OR “adiposity” OR “weight change” AND “Cancer, prostate,” OR “Prostate cancer,” OR “prostate carcinoma” OR Carcinoma. The bibliographic sources of the selected articles were also screened.

### 2.3. Inclusion and exclusion criteria

All the published articles were reports with a description of impact of BMI on prostate cancer, measuring BMI at the beginning and then monitoring the incidence of prostate cancer over time, original research articles with all study designs, and articles published in English were included in this review. Studies that evaluated other than prostate cancer, assessing the impact of other comorbidities in breast cancer patients other than BMI, gray literature, including presented abstracts, letters to the editors, commentaries, systematic review or meta-analysis articles and unavailability of the full text of the article were excluded from the review.

### 2.4. Article screening

Relevant articles were chosen for full-text screening after application of the eligibility criteria. Two authors independently performed the articles screening process and eligibility assessment. In case of some contradictions between the authors, the decision was made by an unbiased third party. The articles were initially screened on the basis of their title, followed by the abstract of the article. In case, the title and abstract of the articles were irrelevant to the present investigation; these were excluded from the secondary screening.

### 2.5. Data extraction

The followed data were extracted from the selected articles that included: first author, year of publication, country, study design, sample size/gender, age, type of prostate cancer testing, height, weight, BMI, disease stage, confounder adjusted, treatment, follow-up duration, outcome measures and main findings.

### 2.6. Risk of bias assessment

The study quality was independently assessed by 2 reviewers using the Newcastle-Ottawa checklist. Divergences were solved by a discussion with the third reviewer. The Newcastle-Ottawa checklist was used for the quality assessment of studies. The total score ranged from 0 to 9, while scores less than 3, less than 6 and between 7 and 9 were considered as low, moderate and high-risk studies, respectively.

### 2.7. Statistical analysis

The meta-analysis was carried out using Review Manager 5.3 (The Cochrane Collaboration, 2014). The total effect size was calculated using a meta-analysis with a 95% confidence interval (CI). Random-effects modeling and generic inverse variance were used to obtain pooled estimates of odds ratio (OR), hazard ratio (HR), and 95% CI, and forest plots were utilized to present the results. Random effects modeling was used because, regardless of the level of statistical heterogeneity. Due to changes in population and treatment, it’s probable that effects differed between trials. The *I*^2^ statistic was used to analyze the clinical heterogeneity of included studies using the Cochran Q test. Significant heterogeneity was defined as *I*^2^ larger than 50%, and this was discussed accordingly. To identify the source of heterogeneity, sensitivity analysis was performed by assessing the influence of different study features such as sample size, publication year, and menopausal status. Statistical tests were 2-sided, and statistical significance was defined as *P* < .05.

### 2.8. Ethical considerations

No ethical approval or patient consent was required because all analyses will be based on already published studies. To prevent ethical issues with regards to plagiarism and copyrights, the findings from the selected articles were duly paraphrased along with acknowledging the work of the authors via the addition of references.

## 3. Results

### 3.1. Eligible studies

**Figure 1.**
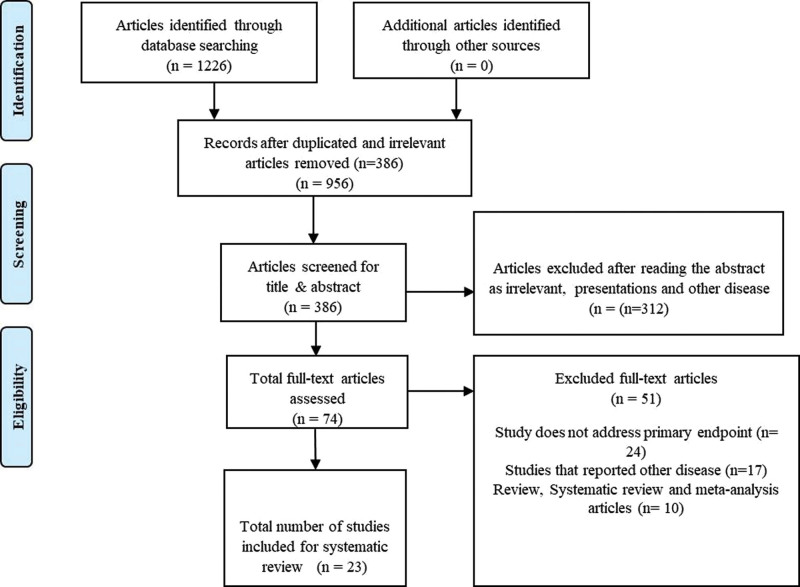
PRISMA chart. PRISMA = preferred reporting items for systematic reviews and meta-analyses.

A total of 1226 articles were found in searched databases, including Cochrane, Google Scholar, and PubMed, of which 840 articles were initially eliminated due to repetition and irrelevance. After analyzing the titles and abstracts at the first screening level, 312 articles were further removed. For full-text evaluations, a total of 74 potential relevant articles were chosen, of which 51 articles were further excluded as studies that reported other cancers and disease (n = 24), studies that related to other comorbidities (n = 17) and review articles (n = 10). Finally, this review included 23 studies that matched the criteria for systematic review inclusion as outlined in the Preferred Reporting Items for Systematic Reviews and Meta-Analyses flow chart (Fig. 1).

### 3.2. Baseline characteristics study

Table 1 shows the baseline characteristics of the studies that were included. Out of 23 included studies, 8 studies were published in United States of America, 5 studies in Sweden, 3 studies in the United Kingdom, whereas, the remaining studies were published in different countries, including Germany, Korea, Denmark, Italy, Australia, Netherland and Norway. In addition, the majority of included studies were prospective cohort studies (n = 17), cohort studies (n = 4) followed by population-based cohort study (n = 1) and retrospective cohort study (n = 1). A total of 2702,312 patients were included in the study, with sample sizes ranging between 668 and 336, 159. All the included patients in this review were male.

**Table 1:** Characteristics of included studies.

S.No	Author & yr	Country	Study design	Sample size/Gender	Age (yrs) Range/Mean/Median	Type of Prostate cancer testing (ng/mL)	Height [HR: 95% conf.interval]	Weight [HR: 95% conf.interval]	BMI range [HR: 95% conf.interval]	Disease stage	Type of analysis	Confounders adjusted	Treatment	Follow up duration	Outcome of measures	Main findings
1	Vidal et al^[17]^	USA	Cohort study	N = 5929/Male	Median = 60	Prostate cancer specific antigen testing, Normal Weight (<25) = 7.1 (5.0, 11.0) Overweight (25 to <30) = 6.5 (4.8, 9.7) Obese (≥30) = 6.1 (4.8, 9.2)	NR	NR	Low or normal = 1.00overweight = 1.16 (0.84–1.62)obese = 1.23 (0.87–1.73)	Early	Multivariate analysis	Age, physical activity, ethnicity, risk factors	NR	7.4 yrs	Weight, BMI, height PSA	Obesity was found to be associated with an increased risk of PCSM
2	Jochems et al^[18]^	Sweden	A prospective cohort study	N = 431902/Male	Mean = 69.2 (8.4)	Prostate cancer specific antigen testing, <4 = 2429 (9) 4-9.9 = 10 818 (39) 10-49.9 = 9402 (34) ≥50 = 4342 (15)	<173 = 1.00173–176 = 1.01 (0.89–1.16)177–180 = 1.00 (0.86–1).16181–183 = 0.96 (0.83–1.12)>/184 = 1.00 (0.85–1.18)	NR	\<22.5 = 0.93 (0.79–1.09)22.5–24.9 = 1.0024–24.9 = 1.20 (0.98-1.46) 25–27.4 = 1.12 (0.98–1.28) 27.5–29.9 = 0.98 (0.81–1.17)>/30 = 0.87 (0.68–1.11)	Early	Cox regression	Age, height, BMI, smoking status	NR	28 yrs	Weight, BMI, height, PSA	BMI was positively associated with PCa-specific mortality
3	Wissing et al^[19]^	Germany	A prospective cohort study	N = 1971/Male	Median = 62	<5.00 ng/mL = 218 (34.4%) 5.00–7.49 ng/mL = 212 (33.5%) ≥7.50 ng/mL = 197 (31.1%)	NR	NR	18.5–24.9 (normal weight) = 220 (34.8%) 25.0–29.9 (overweight) = 312 (49.3%) 30.0–34.9 (obesity class 1) = 73 (11.5%) ≥35.0 (obesity class 2–3) = 8 (1.3%)	Localized	Multivariate analysis	Age, BMI, ethnicity, physical activity	NR	69 mo	BMI, height, PSA	Patients with a higher BMI also had a larger prostate at surgery. BMI and physical activity were not associated with positive surgical margins
4	Dickerman et al^[20]^	USA	A prospective cohort study	N = 5158/Male	Mean = 40–75	Prostate cancer specific antigen testing, <4, % = 124–<10, % = 57–10+, % = 24	N = 69.9 (2.6)	NR	<25 kg/m^2^ = 1.00 (ref) 25–<30 kg/m^2^ = 0.89 (0.71, 1.10) >/30 kg/m^2^ = 1.15 (0.81, 1.65)	Localized	Multivariate analysis	Age, height, BMI, smoking status	Radical prostatectomy, Radiation therapy,	NR	BMI, height, PSA	Metabolic changes associated with weight gain may promote prostate cancer progression.
5	Perez-Cornago et al^[21]^	UK	A prospective cohort study	N = 7024/Male	Mean = 67.8	Prostate cancer specific antigen testing	176.2 cm = 7.2174.3 cm = 7.2172.6 cm = 7.6	71.3 kg = 7.382.7 kg = 7.7 97.3 kg = 11.4	Low or normal = 23.0 (1.6)overweight = 27.2 (1.4)obese = 32.6 (2.7)	Advanced	Multivariate analysis	Age, smoking status, physical activity	NR	13.9 yrs	Weight, BMI, height	BMI was positively associated with prostate cancer death
6	Choi et al^[22]^	Korea	A population based cohort study	N = 139,519/Male	Mean = 40- 64	Prostate cancer specific antigen testing	NR	NR	Low or normal = 0.852 (0.734–0.99)overweight = 1.05 (0.985–1.119)obese = 1.133 (1.067–1.204)	NR	Multivariate analysis	Age, smoking status, Alcohol consumption,	NR	10 yrs	BMI	The hazard ratio (HR) for prostate cancer significantly increased as BMI increases.
7	Cantarutti et al^[23]^	Sweden	Cohort study	N = 3161/Male	Mean = 67	Prostate cancer specific antigen testing, <22.5 = 138 (489.4) 22.5 < 25 = 107 (485.6) 22.5 < 25 = 78 (322.5) 27.5 = 68 (254.7)	<172 cm = 197 (20.6)172–175 cm = 226 (23.7)176–179 cm = 215 (22.5) 180 cm = 316 (33.2)	<22.5 = 66 (6.7) 22.5 < 25 = 75 (5.7) 22.5 < 25 = 82 (6.1) 27.5 = 94 (11.2)	<22.5 = 21 (1.2) 22.5 < 25 = 24 (0.68) 22.5 < 25 = 26 (0.7) 27.5 = 30 (2.7)	Early	Multivariate analysis	Age, height, BMI	Surgery and radiation	11 yrs	BMI, PSA, overall mortality	High BMI was associated with a statistically significant increased risk of prostate cancer specific mortality
8	Møller et al^[24]^	Denmark	A prospective cohort study	N = 26,944/Male	Median = 56	Prostate cancer specific antigen testing	141.5–172.4 cm = 1.00 172.5–176.5 cm = 1.23 (1.08–1.40) 176.8–180.8 cm = 1.23 (1.08–1.41) 181.0–206.0 cm = 1.30 (1.14–1.48)	NR	Low or normal = 1.00overweight = 0.94 (0.85–1.04)obese = 0.86 (0.74–0.99)	Early	Multivariate analysis	Age, risk group(stage, PSA) BMI	NR	15.5 yrs	BMI, height	Obese men with prostate cancer had higher prostate cancer specific mortality
9	De Nunzio et al^[25]^	Italy	A prospective cohort study	N = 668/Male	Median = 68 (62–73)	Non obese = 6.03 (4.69–8.44)Non obese with centraladiposity = 6.46 (4.79–9.21)Obese without centraladiposity = 7.1 (5.11–9.53)Obese with centraladiposity = 6.2 (4.33–8.77)	NR	NR	Non obese = 25.2 ± 2.38Non obese with centraladiposity = 27.3 ± 1.81Obese without centraladiposity = 30.9 ± 1.01Obese with centraladiposity = 33.1 ± 3.16	NR	Multivariate analysis	Age, risk group, PSA, BMI	NR	NR	BMI, % waist	Obesity defined by BMI and WC seems to be associated with prostate cancer (CaP) and, more specifically, with high-grade disease at the time of biopsy
10	Bassett et al^[26]^	Australia	A prospective cohort study	N = 17,045/Male	Mean = 27–76	Prostate cancer specific antigen testing	167.7 cm to <172.6 cm = 0.97 (0.83, 1.13)172.6 to <177.6 cm = 1.04 (0.89, 1.21) 177.6 cm = 1.04 (0.88, 1.23)	73.0 kg to < 79.9 kg = 0.94 (0.81, 1.10) 79.9 to < 87.6 kg = 1.12 (0.96, 1.30) > 87.6 kg = 1.03 (0.87, 1.21)	Low or normal = 0.73 (0.59, 0.91) overweight = 0.98 (0.85, 1.12) obese = 0.96 (0.80, 1.15)	Advanced	Cox regression	Age, stage, BMI	NR	15 yrs	Weight, BMI, height	Weight gain during adult life is associated with increased prostate cancer mortality.
11	Stocks et al^[27]^	Sweden	A prospective cohort study	N = 336,159/Male	Mean = 34.7	Prostate cancer specific antigen testing	<173 = 1.00173–177 = 1.06 (1.01–1.12)177–180 = 1.10 (1.04–1.17)180–184 = 1.16 (1.08–1.23)>/184 = 1.14 (1.06–1.22)	NR	Low or normal = 1.06 (0.98–1.15) overweight = 1.11 (1.03–1.19) obese = 1.05 (0.97–1.12)	Advanced	Multivariate analysis	Age, height, BMI, blood pressure	NR	22.2 yrs	Weight, BMI, height	BMI and Waist circumference (WC) were also associated with high-grade CaP.
12	Wallström et al^[28]^	Sweden	A prospective cohort study	N = 10,564/Male	Mean = 45–73	Prostate cancer specific antigen testing	<170 = 1.00171–174 = 1.20 (0.97–1.49)175–178 = 182 1.11 (0.89–1.38)179–181 = 1.09 (0.85–1.39)>/184 = 1.40 (1.13–1.74)	≤85 = 1.00 86–90 kg = 0.92 (0.74-1.16) 91–95kg = 1.10 (0.89-1.37) 96–101kg = 1.09 (0.87 -1.35) ≥ 102 kg = 1.04 (0.83-1.30)	Under weight = 2.09 (1.03–4.22) Normal = 1.00 overweight = 1.04 (0.90–1.21) obese = 1.04 (0.84–1.30)	Advanced	Multivariate analysis	Age, waist, BMI, diabetes	NR	11.0 yrs	% fat, BMI	Height was positively associated with total and non-aggressive PCa risk where as BMI or body fat percentage, and prevalent diabetes were not associated with PCa risk
13	Hernandez et al^[29]^	USA	A prospective cohort study	N = 83,879/Male	Mean = 45–75	Prostate cancer specific antigen testing	<154 = 1.00154–171.9 = 1.04 (0.95–1.13)172–193.9 = 1.09 (0.99–1.19) ≥ 94 = 1.00 (0.90–1.12)	<66 kg = 1.00 66–67.9 kg = 0.99 (0.91–1.08) 68–69.9 kg = 0.98 (0.89–1.08) ≥ 70 kg = 1.01 (0.92–1.11)	Under weight = 0.87 (0.66–1.17) Normal = 1.00 overweight = 1.04 (0.97–1.11) obese = 0.94 (0.85–1.04)	Early and localized	Cox regression	Age, ethnicity, BMI	NR	9.6 yrs	BMI, weight	Evidence that adiposity, as well as increases in adiposity between younger and older adulthood, may influence the development of prostate cancer.
14	Wright et al^[30]^	USA	A prospective cohort study	N = 287,760/Male	Mean = 50–71	Prostate cancer specific antigen testing	<25 = 1.78 m 25–29.9 = 1.78 m 30–34.9 = 1.78 m 35–39.9 = 1.78 m >/40 = 1.77 m	\<58.6 kg = 1.0 58.7–64.5 kg = 1.01 (0.93–1.10) 64.6–69.9 kg = 0.99 (0.91–1.09) 70–76.7 kg = 0.99 (0.91–1.09) > 76.7 kg = 0.92 (0.84–1.02)	Under weight = 0.95 (0.87–1.04) Normal = 1.00 overweight = 1.01 (0.95–1.09) obese = 0.93 (0.84–1.02)	Advanced	Multivariate analysis	Age, physical activity, BMI	NR	5 yrs	BMI, height, Weight	Higher BMI and adult weight gain increased the risk of dying from prostate cancer
15	Rodriguez et al^[31]^	USA	A prospective cohort study	N = 69,991/Male	Mean = 55-75	Prostate cancer specific antigen testing	<25 = 70 inch 25–27.5 = 70 inch 27.5–30.0 = 71 inch 30.0–35.0 = 70 inch >/35 = 70 inch	NR	Under weight = 1.00 Normal = 1.02 (0.96–1.09) overweight = 0.98 (0.90–1.06) obese = 0.91 (0.75–1.12)	Advanced	Multivariate analysis	Age, smoking status, BMI	NR	2 yrs	BMI, weight	BMI was positively associated with risk of nonmetastatic high-grade prostate cancer
16	Kurahashi et al^[32]^	UK	A prospective cohort study	N = 49 850/Male	Mean = 40–69	NR	<159 = 1.0 cm 160–164 = 0.97–1.76 cm 164–167 = 0.94–1.80 cm > 168 = 0.82–1.66 cm	NR	Under weight = 1.00 Normal = 0.77–1.44 overweight = 0.87–1.65 obese = 0.97–1.76	Advanced	Multivariate analysis	Age, risk factors, BMI	NR	NR	BMI, height	No evidence that BMI is associated with the risk of prostate cancer
17	Engeland et al^[33]^	UK	Cohort study	N = 951 459/Male	Mean = 44. 5	Prostate cancer specific antigen testing	<160 = 0.64 (0.57–0.73)160–169 = 0.89 (0.87–0.92)170–179 = 1.00180–189 = 1.06 (1.03–1.09)>/190 = 1.11 (0.99–1.24)	NR	Under weight = 0.92 (0.78–1.08) Normal = 1.00 overweight = 1.07 (1.05–1.09) obese = 1.09 (1.04–1.15)	Advanced	Cox regression	Age, BMI	NR	21 yrs	BMI, height	The risk of prostate cancer increased by both BMI and height.
18	Lee et al^[34]^	USA	A prospective cohort study	N = 8922/Male	Mean = 67	NR	NR	\<86.4 100 = 1.0086.5-91.4 = 1.30 (0.96-1.76) 91.5-96.5 = 1.31 (0.96-1.80) > 96.5 = 1.19 (0.85-1.65)	Under weight = 1.00 Normal = 1.27 (0.94–1.71) overweight = 1.26 (0.92–1.72) obese = 1.02 (0.68–1.53)	Early	Multivariate analysis	Age, BMI, weight	NR	NR	BMI, Weight	BMI did not yield any significant association with prostate cancer
19	Schuurman et al^[35]^	Netherlands	Cohort study	N = 58,279/Male	Mean = 55- 69	NR	<170 = 1.00170–174 = 0.90 (0.65–1.24)175–179 = 1.08 (0.79–1.47) 180–184 = 0.98 (0.70–1.37)185–189 = 0.78 (0.51–1.19)>/190 = 0.96 (0.52–1.75)	NR	Under weight = 1.00 Normal = 1.20 (0.84–1.73) overweight = 1.35 (0.95–1.90) obese = 0.89 (0.58–1.37)	Advanced	Multivariate analysis	Age, BMI, weight, LBM	NR	6.3 yrs	BMI, height	BMI were not associated with prostate cancer risk
20	Nilsen and Vatten^[36]^	Norway	A prospective cohort study	N = 22,248/Male	Mean = 75.2 (48-96 years)	NR	\<169 = 1.0170–173 = 1.1 (0.9–1.5)174–176 = 1.2 (0.9–1.7)177–180 = 1.3 (0.9–1.8)>/181 = 1.3 (0.9–1.9)	\<69.5 = 1.070.0-75.0 = 0.8 (0.7-1.1)75.5-80.5 = 1.0 (0.8-1.2)81.0-87.0 = 1.1 (0.9-1.4)>/87.5 = 1.0 (0.8-1.3)	\<23.0 = 1.023.1–24.7 = 0.8 (0.6–1.1)24.8–26.2 = 1.0 (0.8–1.3)26.3–28.2 = 0.9 (0.7–1.2)>/28.3 = 1.0 (0.8–1.3)	Advanced	Multivariate analysis	Age, BMI, weight, LBM	NR	12 yrs	BMI, height	Results do not indicate a strong association between body mass index (BMI) and risk of prostate cancer.
21	Giovannucci et al^[37]^	USA	A prospective cohort study	N = 47,781/Male	Median = 20-40	Prostate cancer specific antigen testing	\< 68 inch = 1.0 69 inch = 1.09 (0.91–1.30) 70 inch = 1.07 (0.91–1.27) 71 inch = 1.08 (0.90–1.30) 72 inch = 0.98 (0.80 = 1.19) 73 inch = 1.22 (0.97–1.55) >/ 74 inch = 1.37 (1.10–1.70)	NR	\<23.0 = 1.023–23.9 = 1.25 (1.03–1.51)24–24.9 = 1.20 (0.98–1.46) 25–25.9 = 1.05 (0.87–1.28)26–26.9 = 0.94 (0.74–1.18) 27–27.9 = 1.11 (0.90–1.36)>/29 = 0.90 (0.71–1.15)	Advanced	Multivariate analysis	Age, BMI, weight	NR	2 yrs	BMI, height	Childhood obesity have a strong influence on prostate carcinogenesis.
22	Cerhan et al^[38]^	USA	A prospective cohort study	N = 1050/Male	Mean = 73.5 (65 - 101)	NR	<173 = 1173–177 = 0.8 (0.4–1.4)178–180 = 0.6 (0.3–1.2) >0.80 = 1.1 (0.6–2.0)	< 70.8 = 170.8-77.9 = 1.2 (0.6-2.4)78.0-86.3 = 1.0 (0.5-2.0) > 6.3 = 1.6 (0.8-3.2)	<23.6 = 123.6–25.8 = 0.9 (0.4–1.8)25.9–27.8 = 1.1 (0.6–2.3) >7.8 = 1.5 (0.8–3.0)	Early	Multivariate analysis	Age, smoking status, physical activity	NR	10 yrs	BMI, height, weight	Greater BMI was independent predictors of prostate cancer
23	Andersson et al^[39]^	Sweden	Retrospective cohortstudy	N = 135,049/Male	Mean = 30 - 60	NR	<172 = 1.0172–176 = 1.05 (0.95–1.16)177–180 = 1.07 (0.96–1.21) >80 = 1.14 (1.00–1.29)	<69 = 1.069-75 3 = 1.05 (0.93-1.19)76-82 = 1.04 (0.92-1.17) > 2 = 1.16 (1.03-1.31)	<22.1 = 1.022.1–24.1 = 1.09 (0.94–1.26)24.2–26.2 = 1.10 (0.96–1.26) >6.2 = 1.13 (0.99–1.29)	Early	Multivariate analysis	Age, BMI, weight	NR	20 yrs	BMI, height, weight	The excess risk of death from prostate cancer was statistically significant in all BMI categories

UK = United Kingdom, USA = United States of America.

### 3.3. Risk of bias

The Newcastle-Ottawa checklist was used for risk of bias assessment of the included studies. The risk of bias assessment for each study is shown in Table 2. Among the assessed 23 studies, 12 studies have a “moderate risk,” whereas the remaining 11 studies have a “low risk.” No study was considered to have a “high risk” of bias.

**Table 2:** Risk of bias assessment the included studies.

S. No	Study	Selection				Comparability	Outcome			Overall score	Risk of bias
		Exposed representation	Non-exposed selection	Ascertainment of obesity	Outcome absent at study start	Adjustment by age and nodal status or stage	Outcome assessment	Follow-up length	Adequacy of follow-up		
1	Vidal et al^[17]^	Y	—	Y	Y	—	Y	Y	Y	6	Moderate
2	Jochems et al^[18]^	Y	Y	Y	Y	—	Y	Y	—	6	Moderate
3	Wissing et al^[19]^	Y	Y	Y	Y	Y	Y	Y	—	7	Low
4	Dickerman et al^[20]^	Y	Y	—	Y	Y	Y	—	—	5	Moderate
5	Perez-Cornago et al^[21]^	Y	Y	—	Y	Y	Y	Y	—	6	Moderate
6	Choi et al^[22]^	Y	Y	—	Y	Y	Y	Y	—	6	Moderate
7	Cantarutti et al^[23]^	Y	Y	Y	Y	Y	Y	Y	—	7	Low
8	Møller et al^[24]^	Y	Y	Y	Y	Y	Y	Y	—	7	Low
9	De Nunzio et al^[25]^	Y	Y	Y	Y	Y	—	—	—	5	Moderate
10	Bassett et al^[26]^	Y	Y	Y	Y	Y	Y	Y	—	7	Low
11	Stocks et al^[27]^	Y	Y	—	Y	Y	Y	—	Y	6	Moderate
12	Wallström et al^[28]^	Y	Y	Y	Y	Y	Y	Y	—	7	Low
13	Hernandez et al^[29]^	Y	Y	Y	Y	—	Y	Y	—	6	Moderate
14	Wright et al^[30]^	Y	Y	Y	Y	Y	Y	Y	—	7	Low
15	Rodriguez et al^[31]^	Y	Y	Y	Y	Y	Y	Y	—	7	Low
16	Kurahashi et al^[32]^	Y	Y	Y	Y	Y	Y	—	—	6	Moderate
17	Engeland et al^[33]^	Y	Y	Y	Y	Y	Y	Y	Y	8	Low
18	Lee et al^[34]^	Y	Y	Y	Y	—	Y	—	—	5	Moderate
19	Schuurman et al^[35]^	Y	Y	—	Y	Y	Y	Y	—	6	Moderate
20	Nilsen and Vatten^[36]^	Y	Y	Y	Y	—	Y	Y	—	6	Moderate
21	Giovannucci et al^[37]^	Y	Y	Y	Y	Y	Y	Y	—	7	Low
22	Cerhan et al^[38]^	Y	Y	Y	Y	Y	Y	Y	—	7	Low
23	Andersson et al^[39]^	Y	Y	Y	Y	Y	Y	Y	Y	8	Low

### 3.4. Impact of BMI on prostate cancer

All the included 23 studies were included for meta-analysis to assess the impact of BMI on prostate cancer. Among the selected 23 studies, the BMI data were classified into 3 groups, including Obese (BMI > =30), overweight (25 < BMI < 30 kg/m^2^) and normal weight (<25 kg/m^2^). The effect of BMI on prostate cancer was assessed in selected studies using the OR and HR. The findings of using the random effects approach to analyze studies revealed that they were heterogeneous.

#### 3.4.1. Overweight vs normal weight.

**Figure 2.**
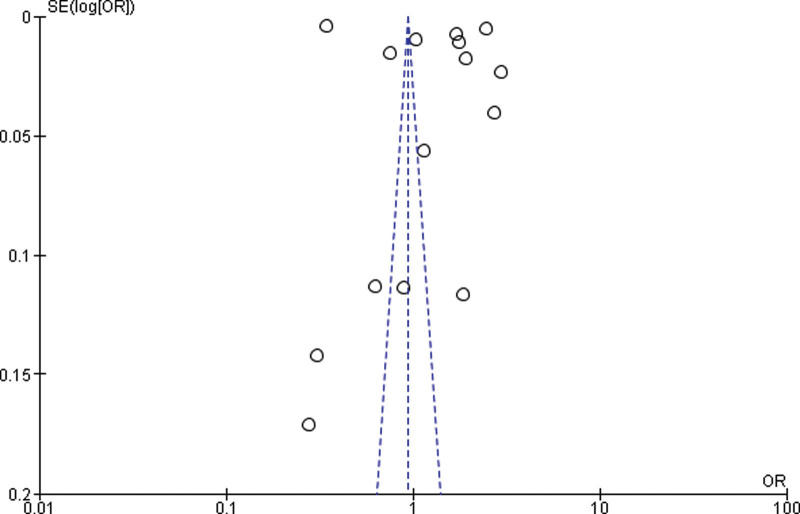
Funnel plot of odds ratio estimates of prostate cancer by overweight versus normal weight.

Fifteen studies were included for the Meta analysis for prospective cohort study and retrospective cohort for normal, overweight and obesity. The study reported that there was no significant difference between the overweight and normal weight (*P* = .75). The study reported that overweight was significantly higher compared with the normal weight. Heterogeneity between the fifteen studies was high (*I*^2^ = 100%). Test for overall effect: Z = 0.31 (*P* = .75) (OR = 1.08 CI: 0.66–1.79) (Table 3 and Fig. 2).

**Table 3:**
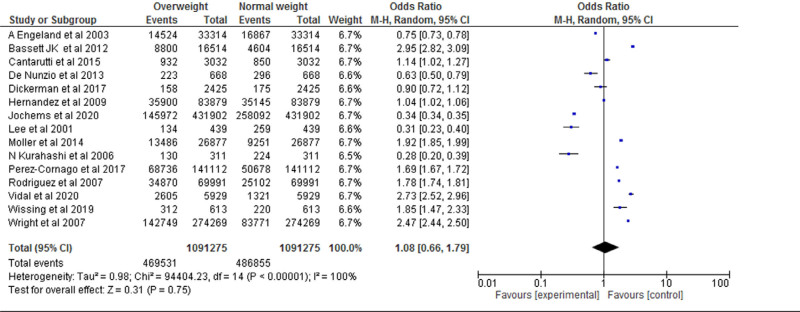
Forest plot for the association between BMI (overweight vs normal weight) and prostate cancer (odds ratio).

#### 3.4.2. Sub-group analysis.

**Figure 3.**
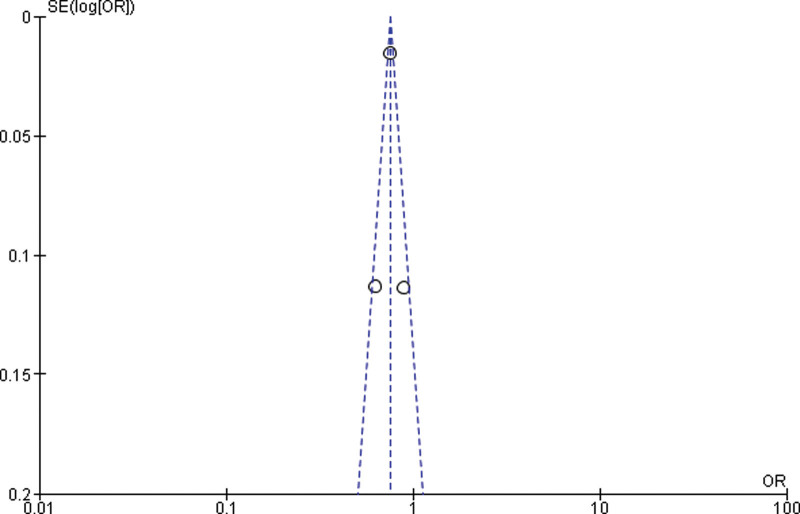
Funnel plot of odds ratio estimates of prostate cancer by overweight versus normal weight after removing 12 studies.

Twelve studies were outside the funnel, so we removed those studies and by rerunning the analysis, we got 3 studies for the Meta analysis between normal weight and overweight. A significant difference between overweight and normal weight (*P* < .001) was found in the study (p0.001). The study reported that obese was significantly higher in the overweight than normal weight (*P* < .001). Heterogeneity between the 3 studies was medium (*I*^2^ = 59%). Test for overall effect: Z = 3.96 (*P* < .0001) (OR = 0.75 CI: 0.65–0.87) (Table 4 and Fig. 3).

**Table 4:**
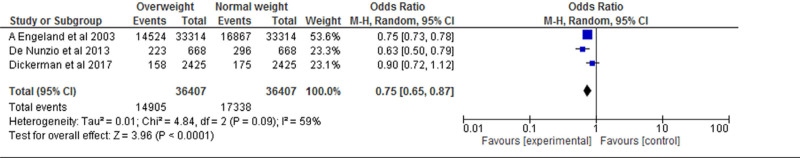
Forest plot for the association between BMI (overweight vs normal weight) and prostate cancer (odds ratio) after removing 12 studies.

#### 3.4.3. Obese versus normal weight.

**Figure 4.**
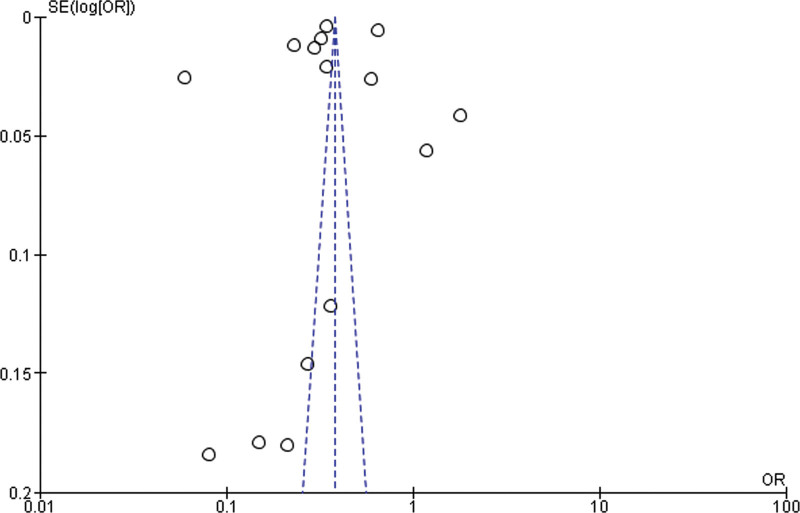
Funnel plot of odds ratio estimates of prostate cancer by obese versus normal weight.

Fifteen studies were included for the Meta analysis of prospective cohort study and retrospective cohort for normal, overweight and obesity. The study reported that there was a significant difference between the normal weight and obese (*P* < .001). The study reported that obese was significantly higher compared with the normal weight. Heterogeneity between the fifteen studies was high (*I*^2^ = 100%). Test for overall effect: Z = 8.77 (*P* < .001) (OR = 0.32 CI: 0.25–0.42) (Table 5 and Fig. 4).

**Table 5:**
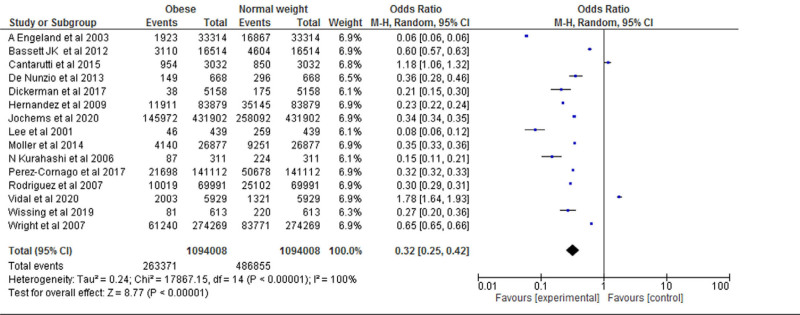
Forest plot for the association between BMI (obese vs normal weight) and prostate cancer (odds ratio).

#### 3.4.4. Sub-group analysis.

**Figure 5.**
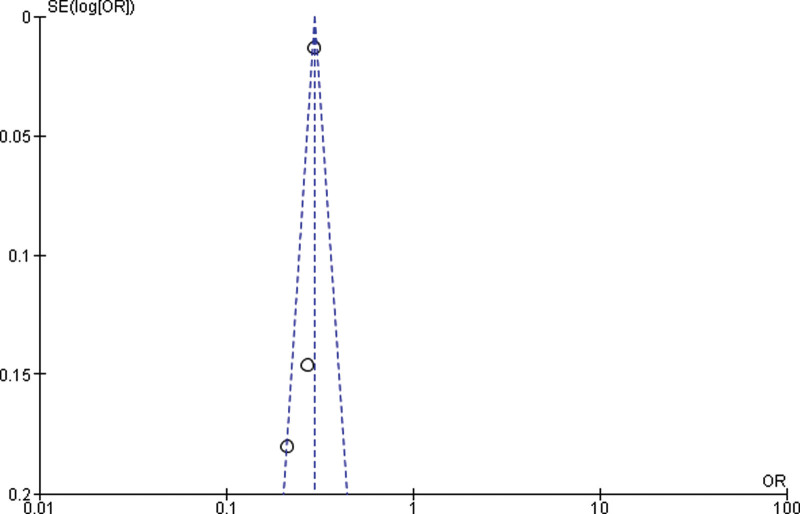
Funnel plot of odds ratio estimates of prostate cancer by obese versus normal weight after removing 12 studies.

Twelve studies lay outside the funnel, so we removed those studies and by rerunning the analysis, we got 3 studies for the Meta analysis of obese and normal weight. The study reported that there was a significant difference between normal weight and obese (*P* < .001). The study reported that obese was significantly higher in the obese compared to normal weight (*P* < .001). Heterogeneity between the 3 studies was medium (*I*^2^ = 51%). Test for overall effect: Z = 14.37 (*P* < .0001) (OR = 0.28 CI: 0.23–0.33) (Table 6 and Fig. 5).

**Table 6:**
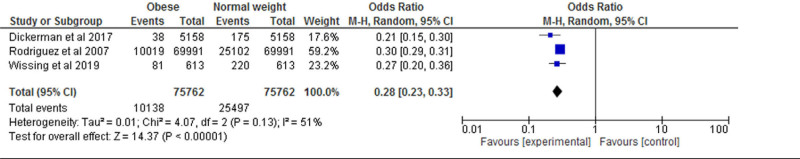
Forest plot for the association between BMI (obese vs normal weight) and prostate cancer (odds ratio) after removing 12 studies.

### 3.5. Hazard ratio

**Figure 6.**
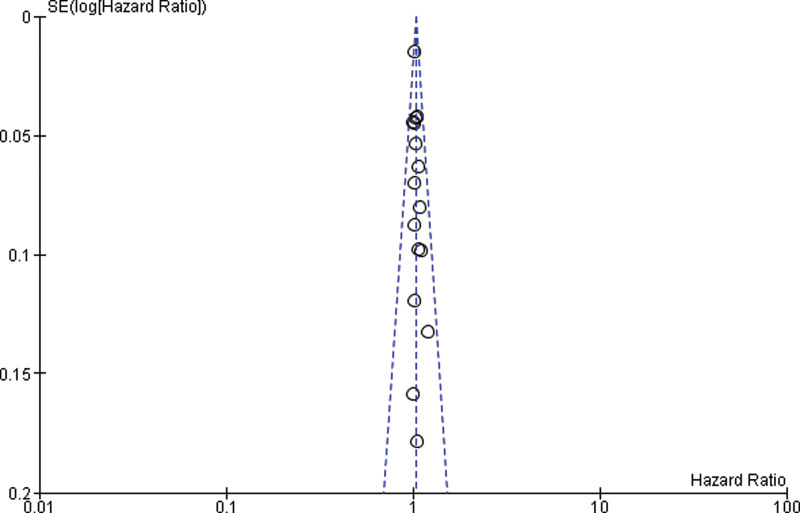
Funnel plot of hazard ratio estimates of prostate cancer by obese versus normal weight.

Sixteen studies were included for the Meta analysis of prospective cohort study and retrospective cohort for normal, overweight and obesity. The study reported that there was significant difference between the normal weight and obese (*P* = .009). Heterogeneity between the fifteen studies was high (*I*^2^ = 0%). Test for overall effect: Z = 2.59 (*P* = .009) (HR = 1.03 CI: 1.01–1.05) (Table 7 and Fig. 6).

**Table 7:**
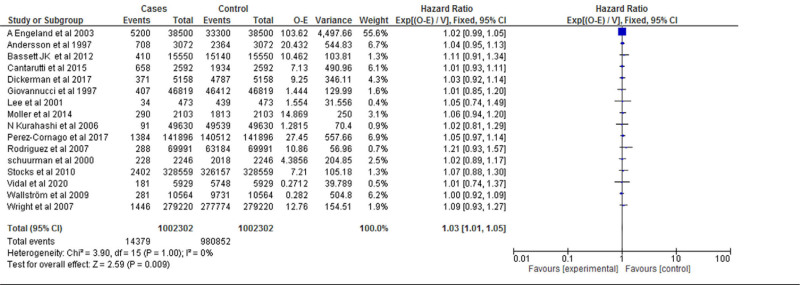
Forest plot for the association between BMI (obese vs normal weight) and prostate cancer (hazard ratios).

## 4. Discussion

Prostate cancer is the second most frequent cancer among men throughout the world.^[7]^ The link between BMI and mortality in prostate cancer patients has been associated with a number of causes. In this review, 23 studies comprising 2702,312 patients were included to explore the relationship between the risk of prostate cancer and BMI. There was no significant difference observed between the normal weight and overweight (*P* = .75) (OR = 1.08 CI: 0.66–1.79) and the heterogeneity between the fifteen studies was high (*I*^2^ = 100%). However, after removing the publication bias in sub-group analysis, there was a significant difference between normal weight and overweight (*P* < .001). High BMI at a young age was negatively associated with overall risk of prostate cancer, according to a prospective analysis of 141,896 males in the European Prospective Investigation into Cancer and Nutrition cohort, as well as fatal and advanced illness,^[8]^ which is similar to the findings of a review. In this review, there is no significant difference observed between the normal weight and obese (*P* < .001) (OR = 0.32 CI: 0.25–0.42) and the heterogeneity between the fifteen studies is high (*I*^2^ = 100%). Similarly, after removing the publication bias in sub-group analysis, there was a significant difference between normal weight and obese (*P* < .001). Our study findings are correlated with the study by Mario Rivera-Izquierdo et al,^[16]^ which reported that obesity was associated with increased prostate-specific mortality (HR: 1.19, 95% CI: 1.10–1.28, *I*^2^: 44.4%) and all-cause mortality (HR: 1.09, 95% CI: 1.00–1.18, *I*^2^: 43.9%). Higher BMI was linked to an increased risk of prostate cancer, according to the findings, which were gathered from 23 studies. Recently, a review by Harrison et al^[19]^ observed no evidence of an association between BMI and the risk of prostate cancer, which is a contrast to our present findings. However, a meta-analysis by Cao and Ma^[20]^ detected high heterogeneity among the studies reviewed in the present analysis. The stratified meta-analyses revealed a clear and persistent link between BMI and increased prostate cancer mortality. Similarly, a meta-analysis of 13 advanced prostate cancer studies and 12 prostate cancer studies found that localized prostate cancer had an inverse linear connection with BMI and advanced prostate cancer had a positive linear link with BMI.^[15]^

The present review has some limitations. Firstly, we used subgroup analysis to find the major source of heterogeneity after observing it across studies. Secondly, the lack of data of confounding factors in the analysis of data such as cigarette smoking or age. Thirdly, unmeasured variables linked to BMI may have altered the outcomes of individual research, even though many of the studies had adjusted for key risk factors. Despite these limitations, this updated systematic review and meta-analysis provides an evidence-based report on the impact of BMI on prostate cancer demonstrated by pooled effect of different studies using rigors methodology.

## 5. Conclusion

In conclusion, according to the findings of the studies examined, a greater BMI is linked to a higher risk of prostate cancer. Future studies will evaluate the influence of BMI on patient mortality in prostate cancer patients should be stratified by cancer type, with well-controlled confounding variables including disease duration, therapy, and lifestyle. Furthermore, wherever possible, the impact of weight change on prostate cancer patient mortality should be investigated.

## Author contributions

**Data curation:** Mohamed Chahine.

**Methodology:** Mohamed Chahine.

**Writing – original draft:** Nikolaos Tzenios.

**Writing – review & editing:** Mary E. Tazanios.
